# Sensitivity and specificity of PS/AA-modified nanoparticles used in malaria detection

**DOI:** 10.1111/1751-7915.12021

**Published:** 2013-06-11

**Authors:** Raweewan Thiramanas, Kulachart Jangpatarapongsa, Udom Asawapirom, Pramuan Tangboriboonrat, Duangporn Polpanich

**Affiliations:** 1 National Nanotechnology Center, National Science and Technology Development AgencyThailand Science Park, Pathum Thani, 12120, Thailand; 2 Center for Innovation Development and Technology Transfer, Faculty of Medical Technology, Mahidol UniversityBangkok, 10700, Thailand; 3 Department of Chemistry, Faculty of Science, Mahidol UniversityPhyathai, Bangkok, 10400, Thailand

## Abstract

Polystyrene (PS) nanoparticle (NP) copolymerized with acrylic acid (AA) and coloured monomer, i.e. 2,3,6,7-tetra(2,2′-bithiophene)-1,4,5,8-naphthalenetetracarboxylic-*N*,*N′*-di(2-methylallyl)-bisimide (ALN8T), was synthesized via the miniemulsion polymerization. Before applying for malaria antigen detection, the blue NP was conjugated with human polyclonal malaria IgG antibody (Ab) specific to *Plasmodium falciparum.* For the conjugation, three methods, i.e. physical adsorption, covalent coupling and affinity binding via streptavidin (SA) and biotin interaction, were employed. The optimum ratio of Ab to NPs used in each immobilization procedure and the latex agglutination test based on the reaction between Ab conjugated NPs and malaria patient plasma were investigated. All Ab–latex conjugates provided the high sensitivity for the detection of *P. falciparum* malaria plasma. The highest specificity to *P. falciparum* was obtained from using Ab–NPs conjugated via the SA–biotin interaction.

## Introduction

Malaria is one of the largest diseases in the developing tropical countries including Thailand. Among the five main species, i.e. *Plasmodium (P.) falciparum*, *P. vivax*, *P. Malariae*, *P. ovale* and *P. knowlesi*, *P. falciparum* is the most important parasite causing morbidity and mortality (Wongsrichanalai *et al*., 2007). Although various anti-malarial drugs are commercially available, the drug resistance still becomes a major concern due to the indiscriminate dispensing of the most effective drug and the parasite diagnosis (Hauck *et al*., 2010). The microscopic examination of stained blood smear is a high sensitive and specific technique which provides a clear identification of parasite stages, i.e. ring, trophozoite, schizont and gametocyte. However, it is labour-intensive, time-consuming and requires considerable expertise (Moody, 2002). Therefore, the rapid antigen detection methods including enzyme-linked immunosorbent assay (ELISA), immunochromatographic test (ICT), and latex agglutination test (LAT), have been developed (Polpanich *et al*., 2007; Murray *et al*., 2008; Martin *et al*., 2009). The LAT, a simple, rapid, and low cost without the need of sophisticated equipment or specialized skill, is of great interest (Bangs, 1996). Its principal involves the reaction between antibody (Ab) immobilized onto the surface of nanoparticles (NPs) and specific antigen in the sample to form network structure of the particles which can be visually observed. Our previous work reported the successful detection of *P. falciparum* via the LAT of Ab-poly(styrene-*co*-acrylic acid) particles with high sensitivity (90%) (Polpanich *et al*., 2007). However, the non-specific agglutination occurred when *P. vivax*-infected plasma greatly concerned. The orientation of Ab bound to NPs might be responsible for the efficiency of the immunoassay (Lazcka *et al*., 2007). In order to attain the high biological activity, the Ab molecule must attach to the particle surface by facing its F(ab′)_2_ fragment to the aqueous phase (Turková, 1999). Since the physical adsorption was used for the Ab attachment, the orientation of Ab on the surface could not be controlled. Moreover, Ab molecules were easily leached out from the NPs surface especially in the presence of surfactant (Peula *et al*., 1998; Nakanishi *et al*., 2008). To solve these problems, the covalent coupling and affinity binding via the streptavidin (SA)–biotin interaction are the better methods of choice.

In this present study, we aim to improve the specificity of the LAT for malaria diagnosis by conjugation of human polyclonal Ab to *P. falciparum* malaria onto NPs. Three methods, i.e. physical adsorption, covalent coupling, and affinity binding, were used for immobilizing Ab onto NPs of polystyrene (PS) copolymerized with acrylic acid (AA) and a coloured monomer, i.e. 2,3,6,7-tetra(2,2′-bithiophene)-1,4,5,8-naphthalene-tetracarboxylic-*N*,*N′*-di(2-methylallyl)-bisimide (ALN8T) (PS/AA-ALN8T) (Goddard and Hotchkiss, 2007; Saerens *et al*., 2008; Polpanich *et al*., 2011). The immunological reactivity of the blue Ab–NPs for capturing *P. falciparum* and *P. vivax* patient plasma was observed via naked eyes. Sensitivity and specificity of the technique were then evaluated. The morphology of immunocomplexes was investigated under optical microscope (OM).

## Results and discussion

### Characteristics of PS/AA-ALN8T

**Figure 1:**
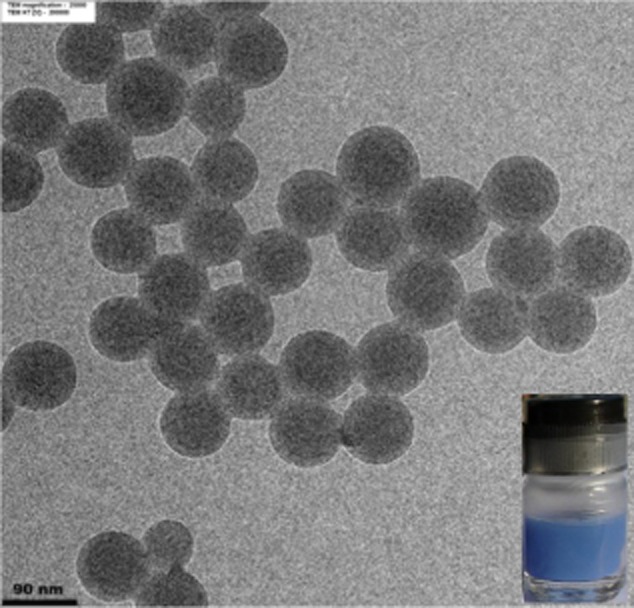
TEM micrograph of PS/AA-ALN8T NPs (Inset is the photograph of the blue latex).

The morphology of PS/AA-ALN8T NPs was investigated under Transmission Electron Microscope (TEM) and the micrograph is displayed in Fig. 1. The photograph of the blue latex captured under daylight is shown as an inset.

The monodisperse and spherical PS/AA-ALN8T particles were clearly observed in Fig. 1. An average size of 92.7 ± 3.9 nm of dried NPs was slightly lower than the D*_h_* determined from the light scattering technique (99.7 ± 0.4 nm) due to the presence of poly(acrylic acid) (PAA) chains at the NPs’ surface. It is well known that the hydrated PAA chains preferred to stay at the particle-water interface and electrosterically stabilized the particle. After polymerization, the coagulum or particle flocculation could not be observed possibly due to the low concentration of SDS (10 mM) and the absence of free PAA chains in the aqueous phase (Nambam and Philip, 2012). It was worth mentioning that the polydispersity index (PDI) and zeta potential of the NPs were 0.018 ± 0.015 and −48.8 ± 2.6 mV (at pH 5.5) respectively. The results confirmed that the uniform and stable PS/AA-ALN8T NPs were obtained. As shown in the inset, the colour of PS/AA-ALN8T appeared in light blue with the maximum absorbance at 588 nm. Since the ALN8T dye containing allyl groups can copolymerize with St monomer, the dye diffusion out of the particle in the LAT did not take place.

### Characteristics of Ab-PS/AA-ALN8T conjugate

**Figure 2:**
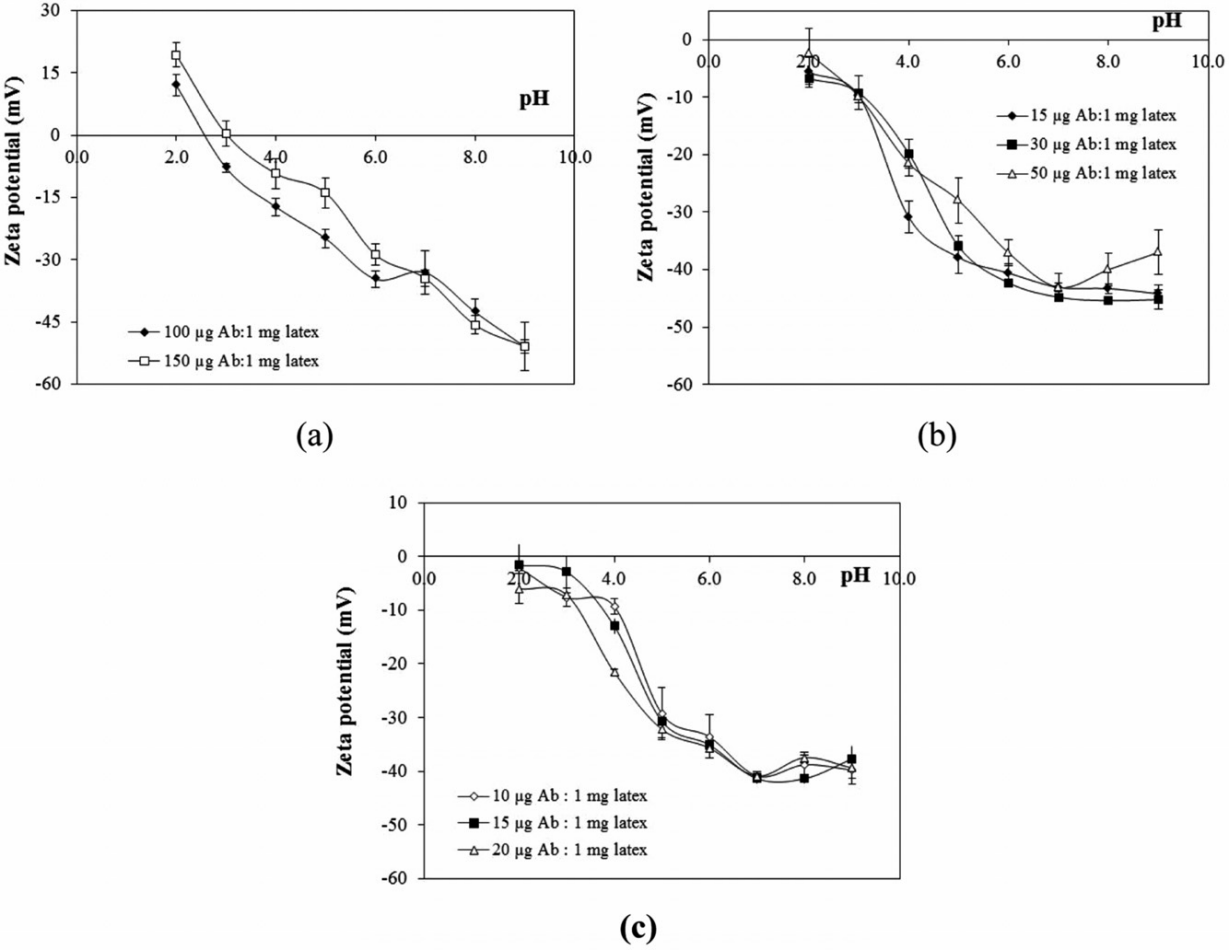
Zeta potential values of (a) p-Ab-PS/AA-ALN8T, (b) c-Ab-PS/AA-ALN8T and (c) aff-Ab-PS/AA-ALN8T, prepared at different ratios of Ab to the latex measured at various pHs (25°C, 1 mM NaCl).

The zeta potentials of polyclonal anti-*P. falciparum* IgG Ab conjugated onto PS/AA-ALN8T via the physical adsorption (p-Ab-PA/AA-ALN8T), covalent coupling (c-Ab-PS/AA-ALN8T) and affinity binding (aff-Ab-PS/AA-ALN8T) at different ratios of Ab to the latex were determined at various pHs (2.0–9.0) and the data are presented in Fig. 2a–c. It should be pointed out that the zeta potential of the as-prepared PS/AA-ALN8T was negative in this pH range due to the presence of-SO_4_^−^ and -COOH derived from potassium persulfate initiator and AA monomer respectively (Polpanich *et al*., 2011; Rahman and Elaissari, 2010).

In Fig. 2a, the zeta potential of p-Ab-PS/AA-ALN8T was positive at pH 2.0 and turned to be negative when increasing pH. The pattern was similar to that obtained from the adsorption of the polyclonal anti-*P. falciparum* Ab onto PS/AA surface (Polpanich *et al*., 2009). It could be assumed that the functional groups at the surface of PS/AA and PS/AA-ALN8T were almost the same even after adding ALN8T in the polymerization reaction. The immobilized amount of Ab at the particle surface, calculated from Eq. 1, was 1.24 ± 0.08 mg m^−2^ (at 150 μg Ab: 1 mg latex). At pH 8.0, the adsorption of negatively charged Ab onto the -COO^−^ and -SO_4_^−^ of PS/AA-ALN8T was caused from the hydrophobic interaction (pI of polyclonal anti-*P. falciparum* Ab was 6.83 ± 0.01). When both Ab and polymer particle carried the same charges, the hydrophobic Fc fragment of Ab approached the particle surface and its hydrophilic F(ab′)_2_ region having antigen binding site faced to the aqueous phase. Thus, the immunoreactivity of the conjugated particles could be enhanced (Dávalos-Pantoja *et al*., 2001). Furthermore, pI of p-Ab-PS/AA-ALN8T shifted to that of the polyclonal anti-*P. falciparum* Ab. A similar effect was found when BSA was adsorbed onto the surface of silica colloidal particles (Rezwan *et al*., 2005). On the other hand, pI of c-Ab-PS/AA-ALN8T and aff-Ab-PS/AA-ALN8T could not be detected and their zeta potential value insignificantly changed over a wide range of pH when varied the amount of Ab used in the immobilization process as illustrated in Fig. 2b and c. It might be due to the low amount of Ab on the NP surface, i.e. 0.21 ± 0.02 and 0.07 ± 0.01 mg m^−2^ for c-Ab-PS/AA-ALN8T (at 50 μg Ab: 1 mg latex) and aff-Ab-PS/AA-ALN8T (at 20 μg Ab: 1 mg latex) respectively. The zeta potential and pI of alumina, titania and zirconia particles could be modulated only when the concentration of lysozyme was > 2 mg m^−2^ (Rezwan *et al*., 2005). Unlike the physical binding, the covalent coupling of Ab onto PS/AA-ALN8T required the reaction of -COOH of AA and EDC to generate an amine-reactive *O*-acylisourea intermediate which was very unstable and susceptible to hydrolysis. Thus, the sulfo-NHS was added to stabilize the intermediate by converting to a semistable amine-reactive NHS ester which subsequently reacted with amine containing Ab to produce amide bond (Gao and Kyratzis, 2008). In the case of aff-Ab-PS/AA-ALN8T, the biotinylation of Ab was firstly performed by interacting -COOH of a valeric acid side-chain of biotin and -NH_2_ of lysine of the polyclonal anti-*P. falciparum* Ab without interrupting the SA–biotin binding function (Hermanson, 2008). Since the size of biotin molecule was smaller than that of Ab, the multiple binding of biotin to an Ab (2.21 biotin for each polyclonal anti-*P. falciparum* Ab molecule) was allowed. Then, the attachment of SA onto the PS/AA-ALN8T particle took place by using EDC as a cross-linker and the amount of SA on the particle was found to be 0.73 ± 0.05 mg m^−2^. Although, SA contains four available sites for strong interacting with biotinylated anti-*P. falciparum* IgG Ab (K_a_ = 10^15^/M), all binding sites were not always bound to the analyte. This was probably due to the steric effect of high molecular weight Ab when approached to the SA coated PS/AA-ALN8T particle leading to the low amount of Ab on the aff-Ab-PS/AA-ALN8T compared with the others (Balthasar *et al*., 2005). The absolute zeta potential values of all conjugated latexes of > 30 mV supported their stability at physiological pH (pH 7.4) which would be suitable for performing LAT.

### Latex agglutination test

As previously reported, at pH closes to the physiological pH, Ab molecule is in its natural conformation for recognizing the epitopes of antigen molecule which resulted in an enhancement of the immunological reaction (Ortega-Vinuesa *et al*., 1996). Therefore, the LAT was performed at pH 7.4 by mixing the conjugated PS/AA-ALN8T latex (1%w/v) with *P. falciparum*-(19 samples) or *P. vivax*-(23 samples) infected plasma while the malaria naive plasma (20 samples) acted as the negative control. The results of agglutination, sensitivity and specificity are summarized in Table 1.

**Table 1:** Summary of agglutination results of PS/AA-ALN8T particles conjugated with Ab via the three methods, i.e. physical adsorption (p-Ab-PS/AA-ALN8T), covalent coupling via EDC/NHS (c-Ab-PS/AA-ALN8T) and affinity binding via streptavidin–biotin system (aff-Ab-PS/AA-ALN8T)

Sample	Agglutination results^a^	Number of samples		
		p-Ab-PS/AA-ALN8T	c-Ab-PS/AA-ALN8T	aff-Ab-PS/AA-ALN8T
*P.* *falciparum*-infected plasma	3+	7	6	7
	2+	10	11	8
	1+	2	2	4
	Negative	0	0	0
*P.* *vivax-*infected plasma	3+	8	3	6
	2+	4	8	3
	1+	10	10	13
	Negative	1	2	1
Malaria naive control	3+	6	1	0
	2+	4	0	3
	1+	3	15	8
	Negative	7	4	9
% Sensitivity		100	100	100
% Specificity		18.6	14.0	23.3

a The results were classified as 3+, 2+ and 1+ if agglutination was seen within 30 s, 1 min and 2 min respectively. Negative was defined for no agglutination occurred within 3 min.

It was observed that all *P. falciparum*-infected plasma gave the positive results with different degree of agglutination when tested with the three conjugated latexes, i.e. the sensitivity, calculated from Eq. 2, of 100% was obtained. The degree of agglutination of the *P. falciparum* positive cases did not significantly correlate (*P* > 0.05) to the results obtained from ELISA. However, from the positive correlation coefficient (0.17, 0.01, and 0.40 for p-Ab-PS/AA-ALN8T, c-Ab-PS/AA-ALN8T, and aff-Ab-PS/AA-ALN8T respectively), the trends of optical density from ELISA and of the degree of agglutination were similar. When using *P. vivax*-infected plasma, the positive results at degree of agglutination of 1+, 2+, and 3+ were 10, 4, and 8 cases for p-Ab-PS/AA-ALN8T, 10, 8, and 3 for c-Ab-PS/AA-ALN8T and 13, 3, and 6 for aff-Ab-PS/AA-ALN8T respectively. This might be due to the cross-reactivity of *P. falciparum* and *P. vivax* species and low specificity of polyclonal anti-malaria IgG Ab purified from the *P. falciparum*-infected plasma. The cross-reactivity of Ab among the blood stages of various *Plasmodium* species was well documented (Diggs and Sadun, 1965; Chuangchaiya *et al*., 2010). Evidence from a study in Thailand indicated the Ab cross-reactivity from a single *P. vivax*-infected patient against both the schizont extract from *P. falciparum* parasite and *P. falciparum* merozoite surface protein 119 (Nagao *et al*., 2008). Monoclonal Ab, raised against the recombinant ectoplasmic region of Apical membrane antigen 1 of *P. vivax*, can cross-react with homologues from *P. knowlesi*, *P. cynomolgi*, *P. berghei* and *P. falciparum* (Igonet *et al*., 2007). In addition, occasional cross-reaction of *P. falciparum* cases at high parasite densities was observed in ICT for detecting *P. vivax*-specific lactate dehydrogenase (Gillet *et al*., 2009).

In the naive control cases, although the p-Ab-PS/AA-ALN8T was prepared at pH 8.0 which encouraged the F(ab_2_)′ fragment of Ab to encounter the aqueous phase as mentioned earlier, the number of false-positive results with high degree of agglutination (3+) were still observed (6 from 20 cases). It was stated that upon binding to NP, the loss of secondary structure and the change in the activity of protein could be induced. This significantly impacted on a protein's function and on its interaction with other molecules (Lynch and Dawson, 2008). A large number of false-positive results (15 from 20 cases) with the lowest degree of agglutination (1+) was also found in the c-Ab-PS/AA-ALN8T. However, the aff-Ab-PS/AA-ALN8T presented no false-positive results at degree of agglutination 3+ and the highest number of true-negative results. Percentage specificity to detect malaria disease of the conjugated latexes calculated from Eq. 3, is listed in Table 1. The highest specificity of 23.3% was obtained from using the aff-Ab-PS/AA-ALN8T. This was because Ab molecule correctly oriented via affinity binding by approaching the antigen binding site to the aqueous phase (Bangs, 1996). Similar to the physical adsorption, the orientation of Ab in the case of c-Ab-PS/AA-ALN8T could not be certainly managed. It was explained that when covalent linkage by using EDC/sulfo-NHS as cross-linker was carried out at pH < 8.0 and the random orientation of Ab was established. One or both of their antigen binding sites might be positioned in such a way that the binding of the antigen is sterically hindered (Puertas *et al*., 2010). This possibly led to a non-specific binding and brought about the lowest specificity of c-Ab-PS/AA-ALN8T.

**Figure 3:**
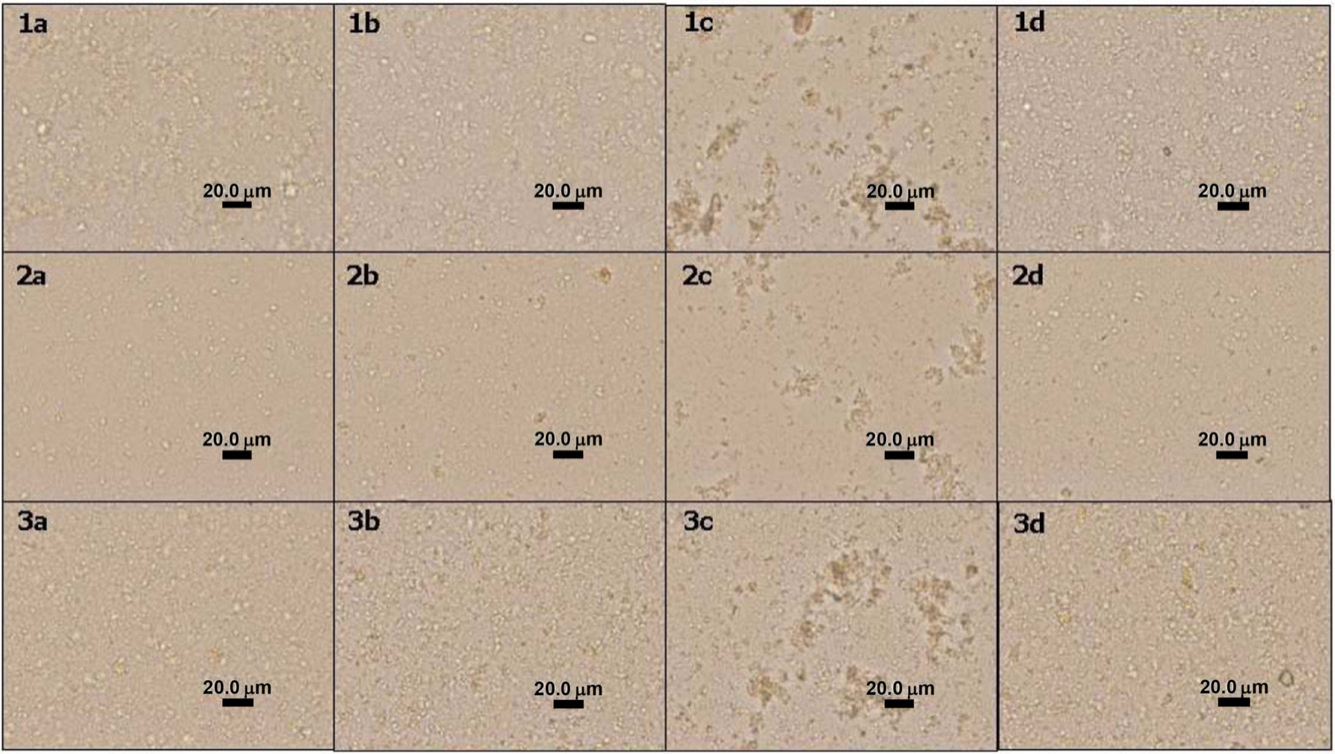
OM images of the immunoagglutination of (row 1) p-Ab-PS/AA-ALN8T, (row 2) c-Ab-PS/AA-ALN8T, and (row 3) aff-Ab-PS/AA-ALN8T (5.0 μl) in the presence of (a) PBS, (b) malaria naive plasma, (c) *P. falciparum*-infected plasma and (d) *P. vivax*-infected plasma (0.5 μl) (mixing time 2 min).

In order to clearly observe the agglutination, the micro-scale agglutinates of the conjugated latexes in the presence of PBS, malaria naive plasma, *P. falciparum-* or *P. vivax*-infected plasma were examined under OM and the images are shown in Fig. 3.

From column c, the agglutinate size of 30 μm was clearly detected by naked eyes. The small aggregates also appeared when p-Ab-PS/AA-ALN8T and aff-Ab-PS/AA-ALN8T were tested with *P. vivax*-infected plasma as shown in column d. However, the pale blue NP did not significantly contribute to the resolution. This might be due to the low amount of ALN8T dye loaded in the PS/AA matrix (0.42%wt based on styrene) which related to the values of 3.63, 6.07, and 3.01 for L*, a*, and b* at 1%w/v. The agglutination of the blue latex on the white card could be obviously observed when their L*, a*, and b* were 48.10, 21.18, and −46.58 respectively (Norrington, 1989).

In summary, this study proposed as an alternative technique for malaria detection. The PS/AA-ALN8T NPs were conjugated with anti-*P. falciparum* IgG by using the three methods, i.e. physical adsorption (p-Ab-PS/AA-ALN8T), covalent coupling via EDC/NHS (c-Ab-PS/AA-ALN8T) and affinity binding via streptavidin–biotin system (aff-Ab-PS/AA-ALN8T). All Ab–latex conjugates showed 100% sensitivity with 19 cases of *P. falciparum*-infected plasma. The highest specificity to *P. falciparum* of 23.3% was obtained from using aff-Ab-PS/AA-ALN8T. Due to the large size of the agglutinate of 30 μm, the NPs could be efficiently visualized via naked eyes. Our findings would be potentially used for the development of a rapid point-of-care diagnostic device for malaria which is suitable for resource-limited or remote area.

## Experimental procedures

### Materials

Styrene (St) (Fluka, Purum) and AA (Aldrich, Anhydrous) were purified by passing through an alumina column and distilled under reduced pressure. A coloured monomer, i.e. 2,3,6,7-tetra(2,2′-bithiophene)-1,4,5,8-naphthalenetetracarboxylic-*N,N’*-di(2-methylallyl)bisimide (ALN8T), was synthesized and characterized as described elsewhere (Polpanich *et al*., 2011). Other chemicals, i.e. potassium persulfate (KPS) (Fluka, Puriss), hexadecane (HD) (Fluka, Purum), SDS (Fluka, BioChemika), ammonium sulfate (Fluka, Purum), 1-ethyl-3-(3-dimethyl aminopropyl)carbodiimide hydrochloride (EDC) (Sigma-Aldrich, Protein sequence grade), *N*-hydroxysulfosuccinimide (sulfo-NHS) (Fluka, Puriss), 2-(*N*-morpholino)ethanesulfonic acid (MES) (Fluka, BioChemika), biotin (Sigma-Aldrich, ≥ 99%), and SA from *Streptomyces avidinii* (Invitrogen), were used without further purification. Deionized water was used throughout this work.

### Synthesis and characterization of PS/AA-ALN8T

The PS/AA-ALN8T latex composing of St, AA and ALN8T was prepared via the miniemulsion polymerization as previously described (Polpanich *et al*., 2011). The hydrodynamic diameter (D*_h_*), PDI and zeta potential of the NPs were measured by using Zetasizer (Nano ZS, Malvern Instruments, UK) at 25°C in NaCl solution (1 mM). TEM (JEM-2010, JEOL, Japan) was used for morphological study. Before protein immobilization, the latex was cleaned by using the dialysis technique (cellulose ester membrane, MWCO 100 kDa). Lightness (L*), hue (a*), and chroma (b*) of the PS/AA-ALN8T NPs were investigated by colorimeter at the wavelength of 380–780 nm using D65 daylight energy distribution (HunterLab, Ultrascan PRO, USA).

### Purification of anti-*P. falciparum* IgG antibody

Plasma samples (20 cases) were collected from acute *P. falciparum*-infected patients who seek for the diagnosis at Malaria Clinic (Mae Sod, Tak province, Thailand). Malaria naive volunteers from non-malaria endemic area were recruited as negative control. This study was approved by the Committee on Human Rights Related to Human Experimentation, Mahidol University. Informed consent was obtained from each individual. High titre plasma (10 cases) determined from ELISA was pooled and proteins were precipitated with saturated ammonium sulfate (Polpanich *et al*., 2007). Then, IgG Ab was purified by Protein G column (Nunc, USA) following the manufacturer's protocol. The concentration and purity of the obtained IgG were examined by the Bradford method (Coomassie® Plus Protein Assay Reagent Kit, Pierce) and SDS-PAGE respectively.

### Conjugation of anti-*P. falciparum* IgG antibody onto PS/AA-ALN8T

Conjugation of Ab onto PS/AA-ALN8T NPs was conducted by using three methods, i.e. (i) physical adsorption, (ii) covalent coupling and (iii) affinity binding.

#### Physical adsorption

PS/AA-ALN8T (0.1 mg) was mixed with the purified polyclonal anti-*P. falciparum* IgG Ab (100 or 150 μg to 1 mg latex). Tris-Cl buffer (0.01 M, pH 8.0) was added to make a final volume of 500 μl. The mixture was incubated at 25°C for 2 h while shaking. After centrifugation at 12 000 r.p.m. for 20 min, the Ab-coated NPs were washed twice and resuspended in PBS buffer (1×, pH 7.4) to obtain 1% solid content.

#### Covalent coupling

PS/AA-ALN8T (0.5 mg) was activated with EDC (2 mM) and sulfo-NHS (5 mM) in MES buffer (25 mM, pH 6.0) containing the anti-*P. falciparum* IgG Ab (15, 30 or 50 μg to 1 mg latex) at 25°C for 3 h. After removing the excess Ab by centrifugation, glycine (50 mM) in MES buffer was added into the mixture for deactivating the residual reactive -COOH groups and blocking the free surface area. The conjugated NPs were washed twice with Tris-Cl (20 mM, pH 9.0) containing 0.1% Tween 20 and, finally, resuspended in PBS buffer (1×, pH 7.4) to obtain 1% solid content.

#### Affinity binding

In this process, biotinylation of the polyclonal anti-*P. falciparum* IgG Ab was first performed. The purified polyclonal Ab (0.5 mg) was dialysed against NaHCO_3_ (0.1 M, pH 8.4) using a dialysis membrane with MWCO 100 kDa (Spectra/Por) at 4°C overnight. Theanti-*P. falciparum* IgG solution was then mixed with freshly prepared biotin solution (1 mg ml^−1^ in dimethylsulphoxide) bringing to the final ratio of 80 μg biotin per mg Ab and incubated at 25°C for 4 h under stirring. The unreacted biotin was removed by dialysis against PBS buffer (1×, pH 7.4) at 4°C for 24 h and, finally, the biotinylated Ab was obtained (Wisdom, 1994). The reacted biotin was quantified by the colorimetric method using biotin quantification kit containing HABA dye (4′-hydroxyazobenzene-2-carboxylic acid) (Pierce).

To conjugate biotinylated anti-*P. falciparum* IgG Ab onto PS/AA-ALN8T by SA–biotin bridge, the latex (0.5 mg) was reacted with SA (50 μg ml^−1^) in the presence of EDC (2 mM) and sulfo-NHS (5 mM) in MES buffer (25 mM, pH 6.0) at 25°C for 3 h. The excess amount of SA was removed by centrifugation and the residual concentration was examined by the Bradford method. After being blocked with glycine (50 mM) in MES buffer (25 mM, pH 6.0) and dispersed in Tris-Cl (20 mM, pH 9.0) containing 0.1% Tween 20, the SA coated NPs were washed with PBS. The biotinylated anti-*P. falciparum* IgG (10, 15 or 20 μg to 1 mg latex) was then added into the latex, the reaction between SA and biotin was allowed to occur at 25°C for 3 h under shaking. The bioconjugated latex was then washed twice and resuspended in PBS buffer (1×, pH 7.4) to obtain 1% solid content (Kokko *et al*., 2007).

The amount of conjugated Ab (Γ) was calculated from the following equation:


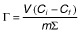
(1)

where *V* (ml) is volume of the solution, *C_i_* (mg ml^−1^) and *C_f_* (mg ml^−1^) are initial and final concentrations of malaria antigen in the solution, respectively, determined by using the Bradford method, *m* (g) is mass of the latex particles, and Σ (m^2^ g^−1^) is specific surface of the PS/AA-ALN8T particles (Revilla *et al*., 1996). Zeta potential of the prepared Ab-PS/AA-ALN8T conjugate was measured at various pHs (2.0–9.0) by using Zetasizer.

### Latex agglutination test

The LAT was simply performed by dropping the Ab-PS/AA-ALN8T latex (1% w/v, 5 μl) onto a glass slide. The plasma from infected patients (*P. falciparum* or *P. vivax*) or malaria naive control (0.5 μl) was subsequently added. After thoroughly mixing on the glass slide for 2 min, the macroscopic agglutination of the NPs was observed by visual inspection. The results were classified as 3+, 2+ and 1+ if agglutination occurred within 30 s, 1 min and 2 min respectively. Sensitivity and specificity were calculated from Eqs 2 and 3 respectively (Al-Yousif *et al*., 2001; Polpanich *et al*., 2007).


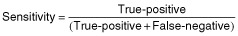
(2)


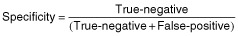
(3)

The morphology of agglutinated particles was investigated under an inverted OM (IX71, Olympus, Japan). Statistical data were analysed using PASW® statistics 18. Correlation was determined by the Spearman rank correlation analysis.
